# Genetic Diversity of “*Candidatus* Liberibacter asiaticus” Based on Four Hypervariable Genomic Regions in China

**DOI:** 10.1128/spectrum.02622-22

**Published:** 2022-11-21

**Authors:** Fanglan Gao, Bo Wu, Chengwu Zou, Yixue Bao, Dean Li, Wei Yao, Charles A. Powell, Muqing Zhang

**Affiliations:** a State Key Laboratory for Conservation and Utilization of Sub-Tropical Bio-Agricultural Resources, Guangxi University, Guangxi, China; b School of Computing, Clemson University, Clemson, South Carolina, USA; c Fruit Experimental Station, Agricultural and Rural Department of Guangxi, Nanning, China; d IRREC, IFAS, University of Florida, Fort Pierce, Florida, USA; USDA–San Joaquin Valley Agricultural Sciences Center

**Keywords:** *Candidatus* Liberibacter asiaticus, huanglongbing (HLB), hypervariable genomic region (HGR), genetic diversity, citrus, hypervariable region

## Abstract

Huanglongbing (HLB; greening disease), caused by *Candidatus* Liberibacter asiaticus (*C*Las), is the most damaging citrus disease worldwide. The disease has spread throughout the citrus-producing regions of Guangxi, Guangdong, Fujian, and others in China. A total of 1,788 HLB-like symptomatic or asymptomatic samples were collected from the Guangxi and Fujian provinces of China to decipher the genetic diversity of *C*Las and its correlation with geographic region and host plant. The disease was the most severe in orange and the least in pomelo. *C*Las bacteria associated with the specific geographical and citrus variety infected more than 50% of the HLB-like symptomatic samples. We identified 6,286 minor variations by comparing 35 published *C*Las genomes and observed a highly heterogeneous variation distribution across the genome, including four highly diverse nonprophages and three prophage segments. Four hypervariable genomic regions (HGRs) were identified to determine the genetic diversity among the *C*Las isolates collected from Guangxi and Fujian, China. A phylogenetic tree constructed from four HGRs showed that 100 *C*Las strains could be separated into four distinct clades. Ten new strains with high variations of prophage regions were identified in the mandarin and tangerine grown in new plantation areas of Guangxi. Characterizing these HGR variations in the *C*Las bacteria may provide insight into their evolution and adaptation to host plants and insects.

**IMPORTANCE** The hypervariable genomic regions derived from 35 published *C*Las genomes were used to decipher the genetic diversity of *C*Las strains and identify 10 new strains with high variations in prophage regions. Characterizing these variations in the *C*Las bacteria might provide insight into their evolution and adaptation to host plants and insects in China.

## INTRODUCTION

Citrus huanglongbing (HLB) is a devastating disease to most citrus species worldwide (1, 2). HLB causes citrus fruit production and quality declines and has severely harmed the citrus industry in China since the disease was discovered in the late 1800s (3, 4). HLB is associated with three Gram-negative, phloem-residing bacteria in the genus *Candidatus* Liberibacter (2, 5 to 9). *Candidatus* Liberibacter asiaticus (*C*Las) widely spread in the main citriculture regions (5); *Ca*. L. africanus (6, 7) and *Ca*. L. americanus (8, 9) are of limited geographic occurrence. Different tolerances to HLB have been observed among citrus varieties, though there has been no natural immunity against the disease in the genus (1, 3). Susceptible citrus trees affected by HLB would develop blotchy mottles on the leaves, stunted yellow shoots, sparse foliation, inward leaf curl with vein corking, and even dieback (1, 3). However, many HLB-affected trees do not show uniform symptoms, and some branches are free of all symptoms (1, 3). Symptom variations on HLB-affected citrus plants were associated with the specific *C*Las populations in Florida. The spatial and temporal variations of different *C*Las populations may contribute to the variations of bacterial titers and HLB symptom expression observed in the infected host plants (2). The genetic diversity of *C*Las populations is deduced from different citrus varieties, geographical origins, population structure, and evolution (10).

The genetic diversity of uncultured *C*Las bacteria is primarily accessed by amplifying the conserved genes, such as the 16S rRNA gene, 16S/23S rRNA intergenic spacer regions (11), the outer membrane protein gene (*omp*) (12), the deoxy-ribonucleotide reductase gene (*nrdB*) (13), the β-operon gene loci (14), the tufB–secE–nusG–rplKAJL–rpoB gene cluster (15), tandem-repeat (16 to 18), or hypervariable prophage regions (19 to 21). Genetic variations within these analyzed nonprophage regions were low and could hardly distinguish closely related *C*Las. *C*Las does have several hypervariable prophage regions (19 to 22). However, the prophage varies from 0 to 2 copies in different *C*Las isolates and could only distinguish partial *C*Las isolates (23 to 25). The publication of over 30 *C*Las genome sequences enables us to find hypervariable genomic regions present in most *C*Las isolates (26 to 28). Such regions will serve as valuable tools in studying *C*Las diversity and potentially associating its pathogenicity with genotypes (29).

One of the most serious concerns for the Guangxi citrus industry is citrus HLB; its incidence level rises quickly (3). Although HLB is an epidemic citrus disease in Guangxi, its citrus production is enlarged and ranked first in China due to the excellent price and extensive domestic requirement (https://www.fas.usda.gov/data/china-citrus-annual-3). Thereby, this research aimed to (i) characterize the new hypervariable genomic regions in the reported genome, (ii) decipher the genomic diversity of *C*Las in a large sample population, and (iii) detect the new strains associated with citrus varieties, symptoms, and geographical locations in Guangxi and Fujian, where most of the intensive citriculture is located, covering different sampling time, geographic locations, and host sources.

## RESULTS

### Genetic variations among 35 *C*Las genomes.

**FIG 1:**
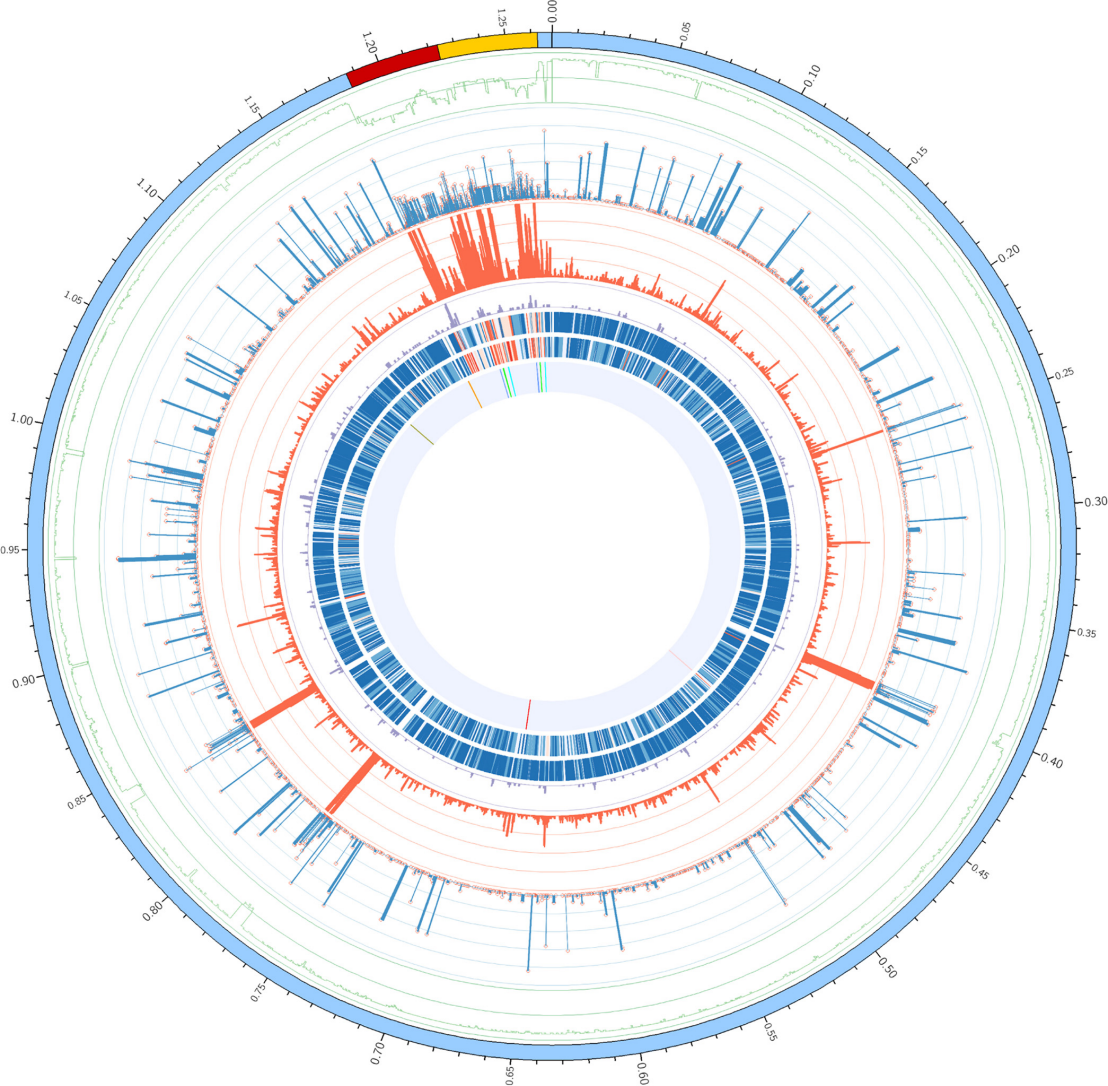
Circular graph depicting the distribution of variants in the 35 published *C*Las genomes. From outer to inner: the ideogram of the reference GXPSY genome, with blue denoting nonprophage regions, red and orange regions denoting the two prophages, and the scale marks (Mb) indicating the coordinates on the reference. The line plot (green) depicts the number of *C*Las genomes in which the homologs of the local 1 kb segments (overlapped by 500 bp) were present; the distribution of the 6,285 variations (red hollow circles) across the reference and their observation times are represented by the height of the lines below the circles in the 35 *C*Las genomes; the two histogram lines show the distributions of SNVs (red) and small indels (violet) in 1-kb windows overlapped by 500 bp across the reference, in which ≥50 and ≥10 values are shown as 50 and 10, correspondingly; the densities of synonymous (outer lane) and missense (inner lane) SNVs in the whole-genome genes are indicated as dark blue (the lowest density) to dark red (the highest density); and the locations of all the segments selected for PCR amplification and genotyping. The four selected segments in the nonprophage region are shown with red dashed lines.

**FIG 2:**
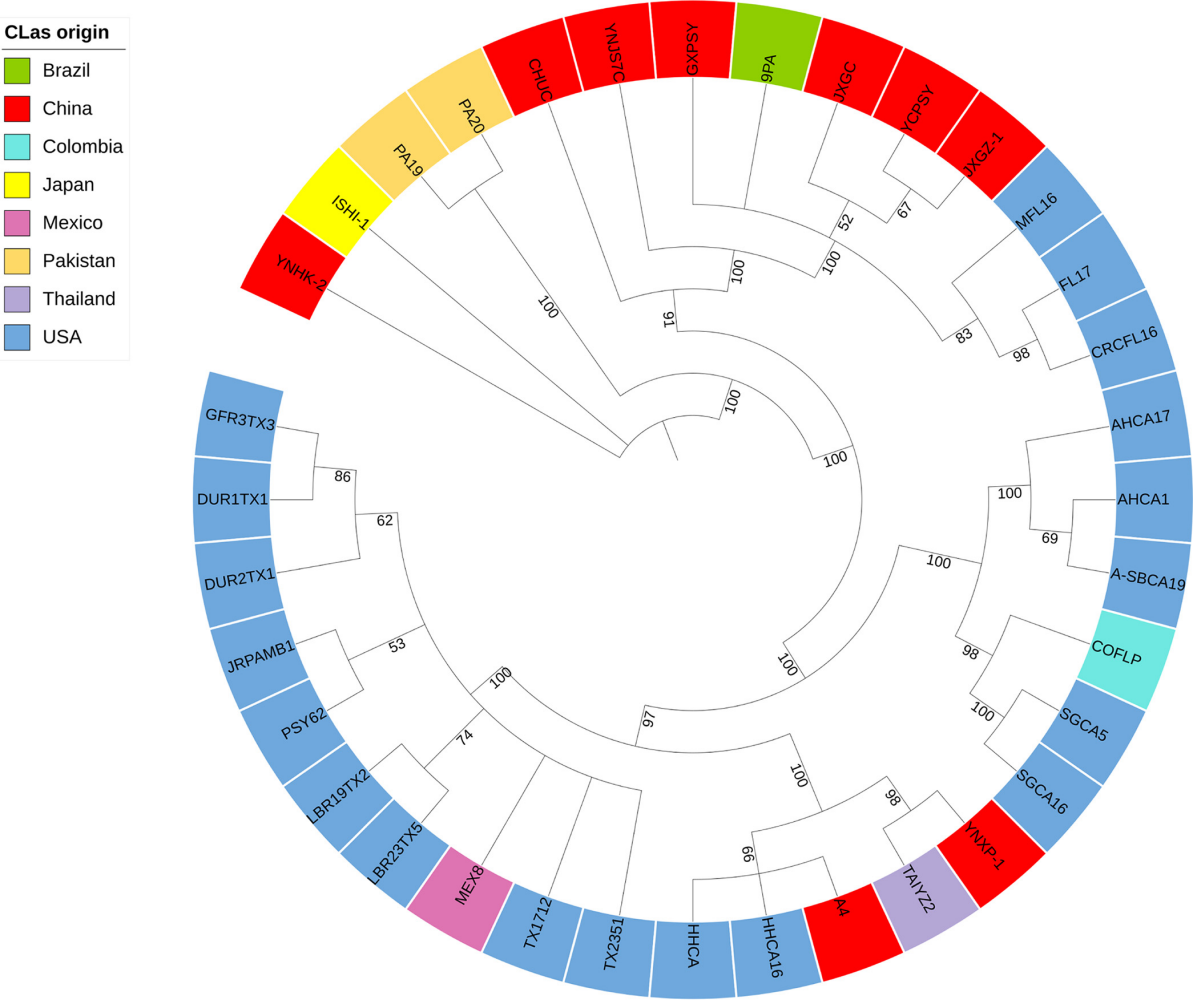
Phylogenetic tree based on 1,954 variations with available genotypes in all *C*Las genomes. The phylogenetic tree was constructed using neighbor-joining (NJ) and Bayesian inference (BI). Only branches with ≥50% bootstrap support (NJ) and with ≥50% posterior probability (BI) are shown in the graph. The numbers on the branches represent the posterior probabilities (%) calculated using Bayesian inference.

To identify genomic segments with high diversity and universal presence in *C*Las isolates, we analyzed 35 published *C*Las genomes from nine countries, including 19 from the United States and 9 from China (Table S1 in the supplemental material). Approximately 14.6% (185 kb) of the reference *C*Las strain GXPSY genome (GenBank accession number CP004005.1, 1,268,237 bp) was missing in at least two genomes, including ~81.5 kb in the prophages and ~103.5 kb in the nonprophage regions (Fig. 1). The genomes had 6,285 small variations, including 6,012 single nucleotide variations (SNVs) and 273 small (≤50 bp) indels (Table S2). The nonreference alleles on 2,160 variations were found in at least two genomes, while the remaining 4,125 were only present in one of the 34 (except GXPSY) genomes. The distributions of SNVs and indels were heterogeneous across the *C*Las genome (Fig. 1). The prophage regions (23.3 variations/kb) had significantly (*P* < 0.001 by the two-tailed *t* test) higher variation density than the nonprophage regions (3.7 variations/kb). Unexpectedly, a high density of variations was found in three duplicate regions harboring rRNA operons in the reference genome located at 398,493 bp to 403,387 bp, 770,848 bp to 775,747 bp, and 838,830 bp to 843,728 bp, most likely due to the assembly errors with next-generation sequencing data. A total of 3,779 variations were annotated in the protein-coding regions, including 992 synonymous, 2,448 missense substitutions, and 117 frameshift indels (Table S3). The prophage genes harbored more synonymous and missense variations (*P* < 0.001) than the non-prophage genes. *C*Las genotyping for collected *C*Las strains was designed based on four nonprophage genomic segments with relatively high genetic diversity and universal presence in the 35 genomes (Fig. 1), including CP004005.1: 1,096,498 to 1,097,081; CP004005.1: 661,737 to 662,418; CP004005.1: 464,946 to 465,629; and CP004005.1: 1,174,796 to 1,175,556. Using neighbor-joining and Bayesian inference, we performed phylogenetic analysis on the 35 *C*Las genomes using 1,954 variations with available genotypes in all genomes. Nine *C*Las strains from China were classified into three distinct clades, at least two of which were introduced into the United States (Fig. 2).

### HLB incidence in citrus groves in Guangxi and Fujian, China.

A total of 1,788 samples were collected in Fujian and Guangxi, including 1,365 (76.34%) being randomly collected from 16 citrus groves and 423 (23.66%) being HLB-like symptomatic leaves from 10 citrus groves. The highest percentage of *C*Las positive in randomly collected samples was 17% in Liuzhou, followed by 14.29% in Baise, 13.49% in Hezhou, and 13.21% in Wuzhou. However, the pathogenic index of *C*Las titers (PI) was over 20% in Wuzhou. HLB-like symptomatic samples had a higher *C*Las positive percentage of 64.77% and a PI of 45.32% than randomly collected samples (Table 1).

**Table 1:** Incidence of *C*Las in prominent citrus-producing areas in Guangxi and Fujian

Sampling method	Sampling sites	Tested samples	*C*Las-positive sample	CLas-positive （%）	*C_T_* value	PI^*a*^ (%)
Random sampling	Yulin, GX	26	3	11.54	38.78 ± 3.37	5.77
	Hezhou, GX	126	17	13.49	39.28 ± 3.27	4.30
	Laibin, GX	65	5	7.69	39.17 ± 3.24	4.62
	Guilin, GX	408	29	7.11	39.51 ± 2.32	2.68
	Wuzhou, GX	106	14	13.21	36.26 ± 5.34	20.83
	Liuzhou, GX	100	17	17.00	38.69 ± 2.99	6.25
	Hechi, GX	100	6	6.00	39.53 ± 2.04	2.50
	Baise, GX	14	2	14.29	38.58 ± 3.51	7.16
	Nanning, GX	203	7	3.45	39.77 ± 1.35	1.23
	Sanming, FJ	217	15	6.91	39.13 ± 3.40	4.44
	Subtotal	1,365	115	8.42	38.79 ± 3.38	5.85
HLB-like sampling	Guigang, GX	75	55	73.33	29.08 ± 6.07	56.25
	Guilin, GX	63	39	61.90	33.55 ± 7.24	33.11
	Yulin, GX	11	11	100.00	30.16 ± 4.11	47.73
	Laibin, GX	15	8	53.33	32.85 ± 7.88	41.67
	Nanning, GX	147	84	57.14	33.76 ± 6.87	31.63
	Liuzhou, GX	26	24	92.31	27.30 ± 5.92	66.35
	Beihai, GX	11	9	81.82	29.20 ± 5.22	56.82
	Sanming, FJ	75	44	58.67	33.00 ± 6.63	36.67
	Subtotal	423	274	64.77	31.16 ± 5.83	45.32

a PI, pathogenic index.

A total of 423 HLB-like symptomatic samples were collected from four citrus varieties, including 136 from mandarin, 64 from orange, 42 from pomelo, 155 from tangerine, and 26 from others. HLB affected all citrus varieties, ranging from 54.76% (pomelo) to 75.00% (orange). PI was highest in orange with the lowest cycle threshold (*C_T_*) value, while it was lowest in pomelo (Table 2). The typical HLB symptoms observed in the HLB-like symptomatic samples included yellowing, blotchy mottle, and Zn-deficient or asymptomatic leaves. Compared to yellowing and asymptomatic samples, blotchy mottle and Zn-deficient samples had a higher *C*Las positive percentage and PI but a lower *C_T_* value (Table 2).

**Table 2:** *C*Las variations in citrus varieties and HLB-like symptom types

Sampling sources	Tested samples	*C*Las-positive (%)	*C_T_* value	PI^*a*^ (%)
Citrus varieties				
Mandarin	136	62.50	33.91 ± 6.06	29.96
Orange	64	75.00	31.03 ± 7.32	44.53
Pomelo	42	54.76	35.84 ± 4.63	19.64
Tangerine	155	57.42	34.05 ± 6.75	29.52
HLB-like symptoms				
Asymptomatic	123	53.66	35.51 ± 5.42	21.95
Yellowing	34	50.00	34.41 ± 6.47	28.68
Blotchy mottle	87	72.41	31.53 ± 6.90	43.10
Zn-deficient	66	83.33	29.25 ± 6.97	55.30
Others	113	64.60	31.98 ± 6.66	38.26

a PI, pathogenic index.

### *C*Las population dynamics in HLB-affected citrus plants in Guangxi and Fujian, China.

Amplicons of the expected size were amplified and sequenced using the seven designed primer sets (PS) (Table 3, Fig. S1). Only a few single-base mutations were detected by PCR amplification using primer sets of PS2, PS3, and PS5 (Fig. S2). The other four primer sets, including PS1, PS4, PS6, and PS7, had high specificity and abundant mutation loci in the amplified regions, making them suitable for genotypic and phylogenetic analysis (Fig. S3). Out of the 343 *C*Las-positive DNA samples from China, 149 samples (43.44%) contained at least one of four hypervariable genomic regions (HGRs) amplified by PS1, PS4, PS6, and PS7, including 120 (80.54%) HGR-I by PS1, 126 (84.56%) HGR-II by PS4, 116 (77.85%) HGR-III by PS6, and 126 (84.56%) HGR-IV by PS7 (Table 4). All amplicons from 100 *C*Las positive samples amplified by all four primer sets were sequenced and aligned. HGR-IV had the most mutation sites, followed by HGR-III, whereas HGR-I was the most conserved region. In the 86 HGR-I sequences amplified by PS1, 25 mutation regions were detected, including 21 six-base deletions and 10 single-base substitutions. For PS4, 37 of the 118 HGR-II sequences had single or multiple base substitutions. For PS6, 73 of the 118 HGR-III sequences had substitutions at 12 sites and one insertion. For PS7, 35 of the 120 HGR-IV sequences had nucleotide substitution, deletion, and insertion mutations (Fig. S3).

**Table 3:** The primer sets (PS) used in this study

Primer sets ID	Primer sequences (5′−3′)	Amplicon size (bp)	*Tm* (°C)	Target region
HLBas/r	F: TCGAGCGTATGCAATACG	100	60	16s rDNA (Li et al., 2006) (33)
	R: GCGTTATCCCGTAGAAAAAGGTAG			
	P: AGACGGGTAGTAACGCG			
PS1	F: GGGCAGAAACAGCAACAGA	583	60	CP004005.1:1096498 to 1097081 in the nonprophage region (this study)
	R: TGTCTCACGCTCTATGGAGGA			
PS2	F: AACGCAAATCGCGTACTCTT	681	60	CP004005.1:661737 to 662418 in the nonprophage region (this study)
	R: TTTTCTGGTTGGGATGTGTG			
PS3	F: GAACAATGCACGCCCTAAAT	683	60	CP004005.1:464946 to 465629 in the nonprophage region (this study)
	R: ATTTGCCACCAAAAGAGAGG			
PS4	F: CAAGAGGCGTATACGGAAGC	760	58	CP004005.1:1174796 to 1175556 in the nonprophage region (this study)
	R: GGGGCAAAGATGAAACTCAA			
PS5	F: AAGCCGATAAAAATGCATGG	798	60	CP004005.1:1215079 to 1215858; CP004005.1:1254946 to 1255744 in two prophage regions (this study)
	R: TGCAATGCGGTAGTTGATGT			
PS6	F: CTTTGTCGTTCCGATCCAAT	580	56	CP004005.1:1220655 to 1221235; CP004005.1:1260522 to 1261102 in two prophage regions (this study)
	R: AAGCGAAAAGGTATCGCAAA			
PS7	F: TGACAGCACGCCTCAATTAC	735	58	CP004005.1:1211371 to 1212109; CP004005.1:1251241 to 125197 in two prophage regions (this study)
	R: TAAAGCCGTTTCCAACTTCG			

**Table 4:** Hypervariable genome regions (HGRs) amplified from the *C*Las strains collected in different hosts, symptoms, and geographical locations in Guangxi and Fujian

Sampling sources	Number of samples	Number and percentage (%) of *C*Las-positive samples				Average amplified (%)
		HGR-I	HGR-II	HGR-III	HGR-IV	
Geographical origins						
Yulin, GX	8	5 (62.50)	8 (100.00)	5 (62.50)	5 (62.50)	71.88
Hezhou, GX	14	12 (85.71)	13 (92.86)	12 (85.71)	12 (85.71)	87.50
Laibin, GX	5	4 (80.00)	5 (100.00)	5 (100.00)	5 (100.00)	95.00
Guilin, GX	16	14 (87.50)	13 (81.25)	13 (81.25)	15 (93.75)	85.94
Wuzhou, GX	1	1 (100.00)	1 (100.00)	1 (100.00)	1 (100.00)	100
Liuzhou, GX	12	10 (83.33)	11 (91.67)	12 (100.00)	12 (100.00)	93.75
Nanning, GX	50	40 (80.00)	39 (78.00)	34 (68.00)	42 (84.00)	77.50
Guigang, GX	23	19 (82.61)	20 (86.96)	19 (82.61)	19 (82.61)	83.70
Beihai, GX	10	7 (70.00)	9 (90.00)	10 (100.00)	9 (90.00)	87.50
Sanming, FJ	10	8 (80.00)	7 (70.00)	5 (50.00)	6 (60.00)	65.00
Hosts						
Tangerine	44	32 (76.19)	40 (90.91)	38 (86.36)	41 (93.18)	86.66
Mandarin	32	26 (81.25)	28 (93.33)	25 (83.33)	26 (86.67)	86.15
Orange	63	55 (90.16)	48 (76.19)	46 (73.02)	52 (82.54)	80.48
Pomelo	10	7 (70.00)	10 (100.00)	7 (70.00)	7 (70.00)	77.50
Symptom types						
Asymptomatic	44	35 (79.55)	36 (81.82)	32 (72.73)	36 (81.82)	78.98
Yellowing	11	9 (81.81)	10 (90.91)	10 (90.91)	10 (90.91)	88.64
Blotchy mottle	30	26 (86.67)	25 (83.33)	26 (86.67)	27 (90.00)	86.67
Zn-deficient like	64	50 (78.13)	55 (85.94)	48 (75.00)	53 (82.81)	80.47
Total	149	120 (80.54)	126 (84.56)	116 (77.85)	126 (84.56)	81.88

HGRs were the least amplified in *C*Las strains collected from Sanming, Fujian, whereas it was highly amplified in those collected from the central parts of Guangxi (Wuzhou, Laibin, and Liuzhou). HGR-II was primarily amplified, while HGR-I, HGR-III, and HGR-IV remained stable in Yulin, Hezhou, and Guigang samples. The amplification rates of HGR-II, HGR-III, and HGR-IV were higher than those of HGR-I in Laibin, Liuzhou, and Beihai samples. More HGR-I and HGR-IV were amplified from the Guilin and Nanning samples than HGR-II and HGR-III. HGR-II and HGR-IV were more amplified in HLB strains from tangerine and mandarin, HGR-I from orange, and HGR-II from pomelo. Regarding citrus leaf symptoms, more HGRs were amplified in samples with typical HLB symptoms (blotchy mottle and yellowing) than in the samples from asymptomatic and Zn-deficient leaves (Table 4).

### Diversity of *Ca*. Liberibacter asiaticus in Guangxi and Fujian, China.

**FIG 3:**
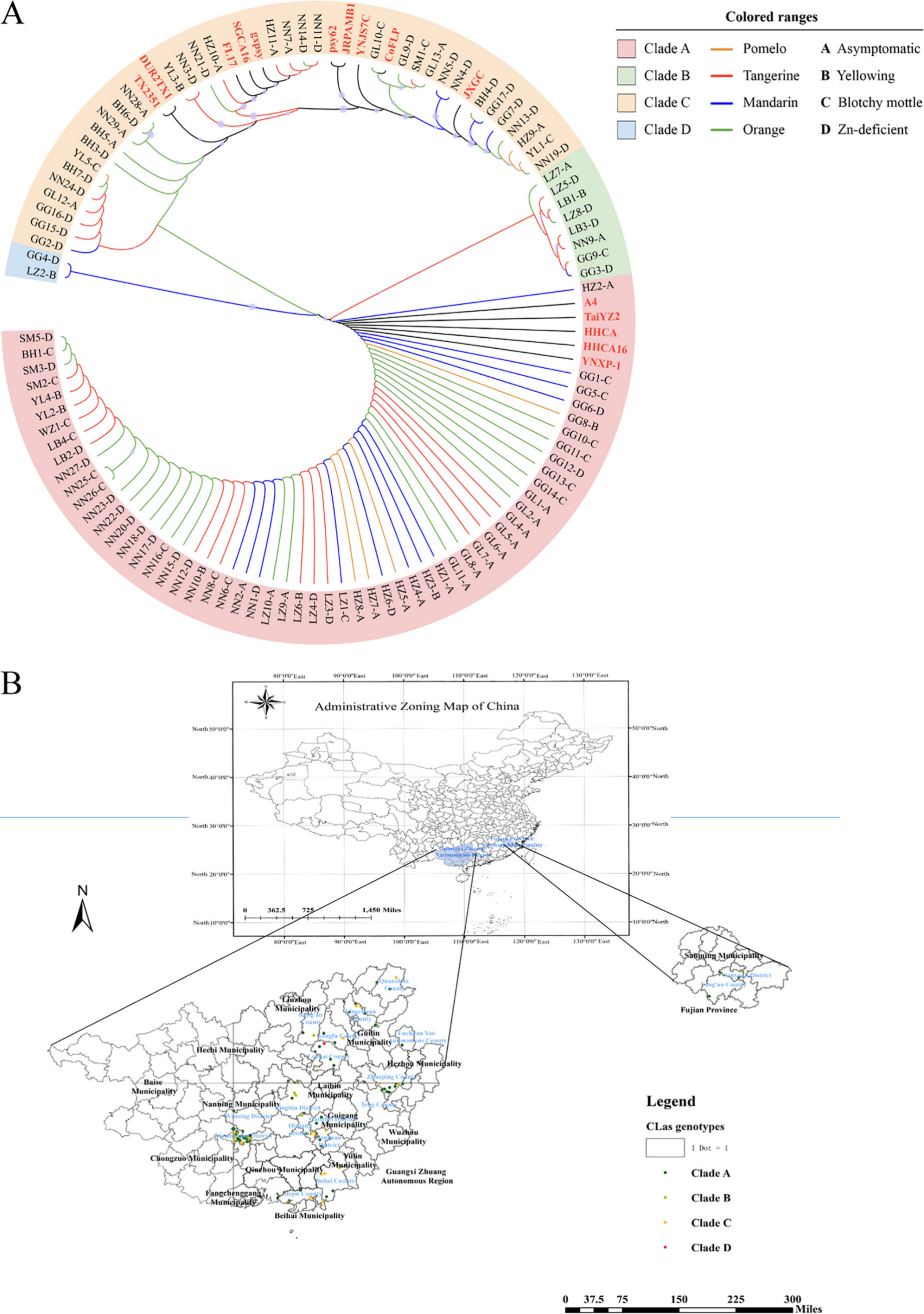
Clustering clade (A) and sampling sites (B) of 100 strains collected from Guangxi and Fujian, China. The maximum likelihood phylogenetic tree in panel A was constructed based on four hypervariable genomic regions of 100 strains and 15 published *C*Las genomes. Bootstrap values expressed as the percentage of 1,000 replications were indicated at the nodes. NTSYS clustering was used to analyze the association of *C*Las strains with the citrus geographical origin, varieties, and HLB symptoms. The map in panel B was drawn with Arcgis 10.2 software.

**FIG 4:**
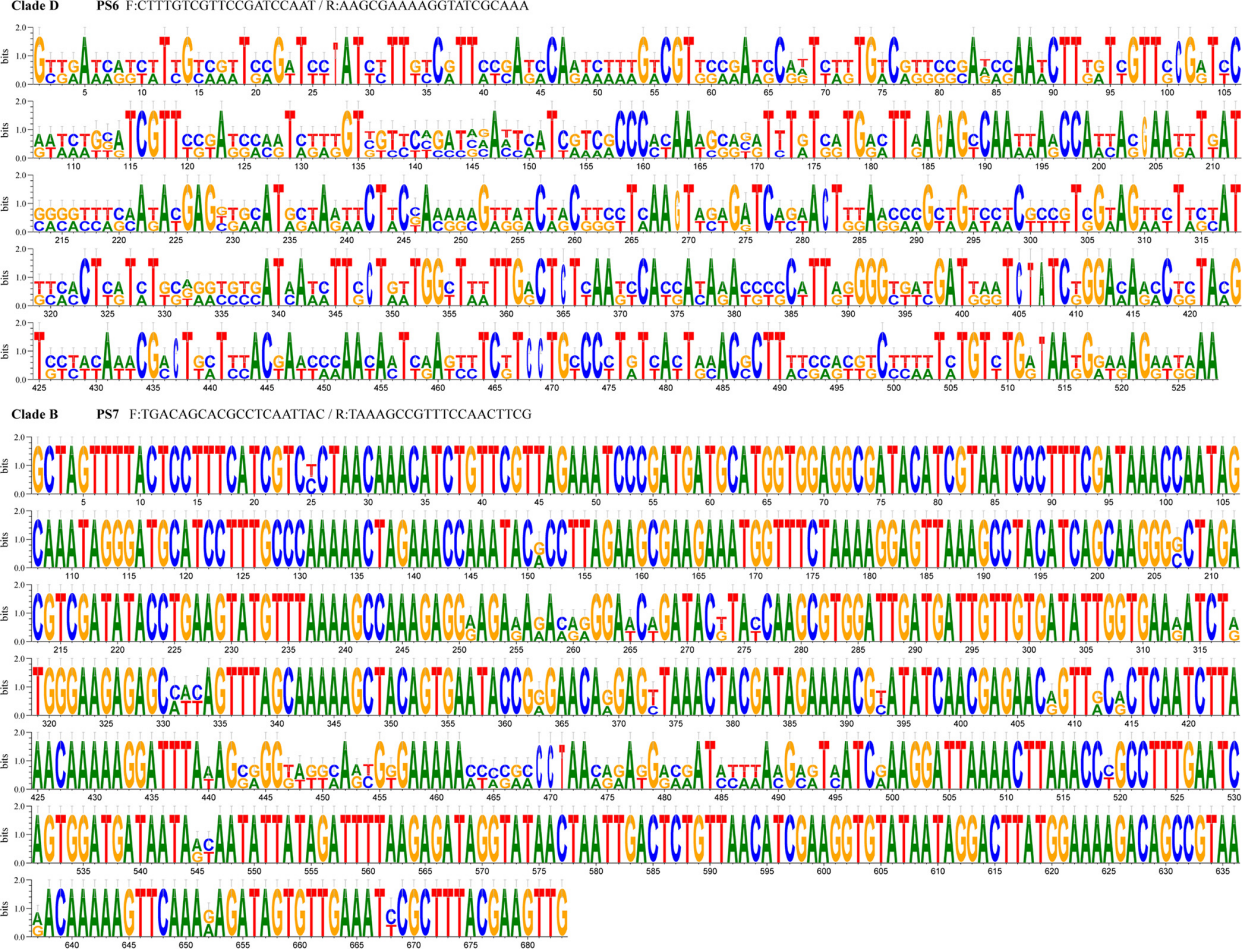
Nucleotide associations in the alignments of 20 amplified sequences from 10 strains in Clade B and Clade D using primer sets of PS6 and PS7 with 15 reported *C*Las downloaded from the NCBI database. The alignments were constructed using DNAMAN.

A total of 228 polymorphic loci were detected from four hypervariable genomic regions (HGRs) of 100 samples, including 74 singleton variable loci and 154 parsimony loci. A phylogenetic tree constructed from the amplified sequences of four HGRs showed that the genetic diversity of *C*Las was not associated with the geographic locations, citrus varieties, and HLB symptoms (Fig. 3). One hundred *C*Las strains were divided into four clades. Fifty-seven strains from different geographic sources and citrus species were classified into Clade A, including five reported TaiYZ2, HHCA, HHCA16, YNXP-1, and A4 isolated from citrus in the United States, Thailand, and China. Thirty-three strains were classified into Clade C, including five reported TX2351, GXPSY, CoFLP, JRPAMB1, and Psy62 from citrus psyllid and five DUR2TX1, SGCA1, YNJSTC, JXGC, and FL17 from citrus, respectively. In Guangxi, the remaining 10 strains collected from newly expanded citrus groves (Liuzhou, Laibing, Nanning, and Guigang) were clustered into two separate clades without reference strains. GG4 and LZ2 from mandarin in Clade D had a high variation in the first 170 bp of the prophage regions amplified by PS6 compared to the 15 reported genomes. The eight strains in Clade B from tangerine were quite different in the prophage regions amplified by PS7, including LZ7, LZ5, LB1, LZ8, LB3, NN9, GG9, and GG3 (Fig. 4).

## DISCUSSION

Citrus HLB is caused by the bacterium *Candidatus* Liberibacter asiaticus (*C*Las) and transmitted by psyllids (1, 2, 30, 31). The genetic diversity of *C*Las from different geographical regions and citrus cultivars is vital to predicting the risk of HLB (10). The incidence of HLB and the distribution of *C*Las were assessed here to determine the potential spread of *C*Las in Guangxi and Fujian, China. The HLB incidence in hot and humid climates was more severe than in cool ones (1, 3). The prevalence of HLB was relatively low in the randomly collected samples from Guilin (the northern part of Guangxi), in regions where the freezing temperatures in winter might inhibit the occurrence and spread of the Asian citrus psyllid (3). The HLB symptoms in the field were diverse, rendering it challenging to distinguish whether *C*Las caused them or not. HLB-like symptomatic samples had a higher *C*Las-positive and pathogenic index of *C*Las titer. Zn-deficient yellowing and blotchy mottle were the most typical HLB symptoms in Guangxi and Fujian, China, accounting for more than 40% of the pathogenic index. The orchards in the valley mainly showed blotchy mottle, while the ones on the ridge performed Zn-deficient yellowing.

The results presented here revealed that new sequences of the hypervariable genomic regions (HGRs) of *C*Las bacteria were derived from the 35 published genomes and used to determine the genetic diversity among the *C*Las isolates collected from Guangxi and Fujian, China. Among the 35 published genomes from nine countries, including 19 from the United States and 9 from China, 185 kb of the reference *C*Las strain GXPSY genome (1.268 Mb) was missing in at least two genomes, including ~81.5 kb in the prophages and ~103.5 kb in the nonprophage regions. The prophage genes had significantly more synonymous and missense variations than the nonprophage genes. Four HGRs with high variations were screened and validated in our strains, including two prophage regions amplified by PS6 (HGR-III, CP004005.1:1220655 to 1221235, and CP004005.1:1260522 to 1261102) and PS7 (HGR-IV, CP004005.1:1211371 to 1212109, and CP004005.1:1251241 to 1251975), and two nonprophage regions amplified by PS 1 (HGR-I, CP004005.1:1096498 to1097081) and PS4 (HGR-II, CP004005.1:1174796 to1175556).

Hypervariable genomic regions (HGRs) resulted in the variation of the *C*Las population in different regions and hosts (10). Our results showed that only 43.44% of the *C*Las-positive samples could amplify at least one of the HGRs, and the amplification frequencies of HGRs differed from each geographical region. HGR-II was primarily amplified in Yulin, Hezhou, and Guigang samples, whereas more HGR-I and HGR-IV were amplified from Guilin and Nanning, Guangxi. Compared with the Guangxi samples, HGRs were the least amplified in CLas strains collected from Sanming, Fujian. HGR-II and HGR-IV were more frequently amplified in the samples from mandarin and tangerine, indicating that different citrus varieties had different HGR amplification frequencies.

The phylogenetic analysis of four HGRs in 100 *C*Las strains demonstrated that the nucleotide sequence of these HGRs allowed our *C*Las strains to be separated into four distinct clades, in which 90% of strains were clustered primarily in two clades with 15 published genomes from Asia and America, and exhibited genetic diversity. JRPAMB1, Psy62, CoFLp, and Gxpsy isolated from citrus psylla clustered in the same clade, while Taiyz2 and A4 isolated from citrus of Thailand and China (in Asia) were separated. The phylogenetic tree confirmed that MEX8 from Mexico was the closest to the U.S. isolates (27). We also found that COFLP from Colombia (28) was related to strains in the United States. Meanwhile, 9PA from Brazil (29) was obtained separately from China or other Asian areas. Ninety *C*Las strains in these two clades had little relationship with geographical growing regions, citrus varieties, and HLB symptoms due to the chaotic nursery stock market in Guangxi, where *C*Las-infected scions were planted without quarantine inspection, accelerating *C*Las transmission (3).

The prophages influence *C*Las pathogenicity, host specificity, and ecological adaptation factors, all of which contribute to the evolution of *C*Las strains (32, 33). The prophages have been found in most sequenced *C*Las genomes, accounting for 6.7% of the *C*Las genome (16, 32). The prophage regions had a higher variation density (23.3/kb) than the nonprophage regions of only 3.7/kb. The results also revealed that 10 strains in the other two clades considerably varied from previously reported *C*Las, which were collected from the newly expanded citrus groves in Liuzhou, Laibing, Nanning, and Guigang of Guangxi. Two strains (GG4 and LZ2) from mandarin in Clade D had a higher variation in the first 170 bp of the prophage regions (CP004005.1:1220655 to 1221235, and CP004005.1:1260522 to 1261102) amplified by PS6. Eight strains (LZ7, LZ5, LB1, LZ8, LB3, NN9, GG9, and GG3) from tangerine in Clade B were quite different from the 15 reported genomes in the other prophage regions (CP004005.1:1211371 to 1212109, and CP004005.1:1251241 to 1251975) amplified by PS7 (Fig. 4). Prophage genes have been demonstrated to be the better markers for differentiating the *C*Las strains from different geographic origins (20, 21, 23). The origin of prophages from Liberibacters associated with plants is not homologous (22, 32, 34).

Because of the consumer demand for a reasonable price and easy-to-peel varieties, more and more tangerine and mandarin are being grown in the new areas of Guangxi, especially the municipalities of Liuzhou, Laibing, Nanning, and Guigang. One of the most serious concerns for the citrus industry in Guangxi is citrus HLB, which is becoming an epidemic and has not been effectively controlled (3). Citrus genotypes have been reported to promote variation in *C*Las strains by deciphering *C*Las populations of various citrus varieties (10). These new strains with high variation in prophage regions were collected from the new plantation areas. Characterization of the variations of these prophages in the *C*Las bacteria may provide insight into their evolution and adaptation to host plants and insects.

## MATERIALS AND METHODS

### Sample collection.

**FIG 5:**
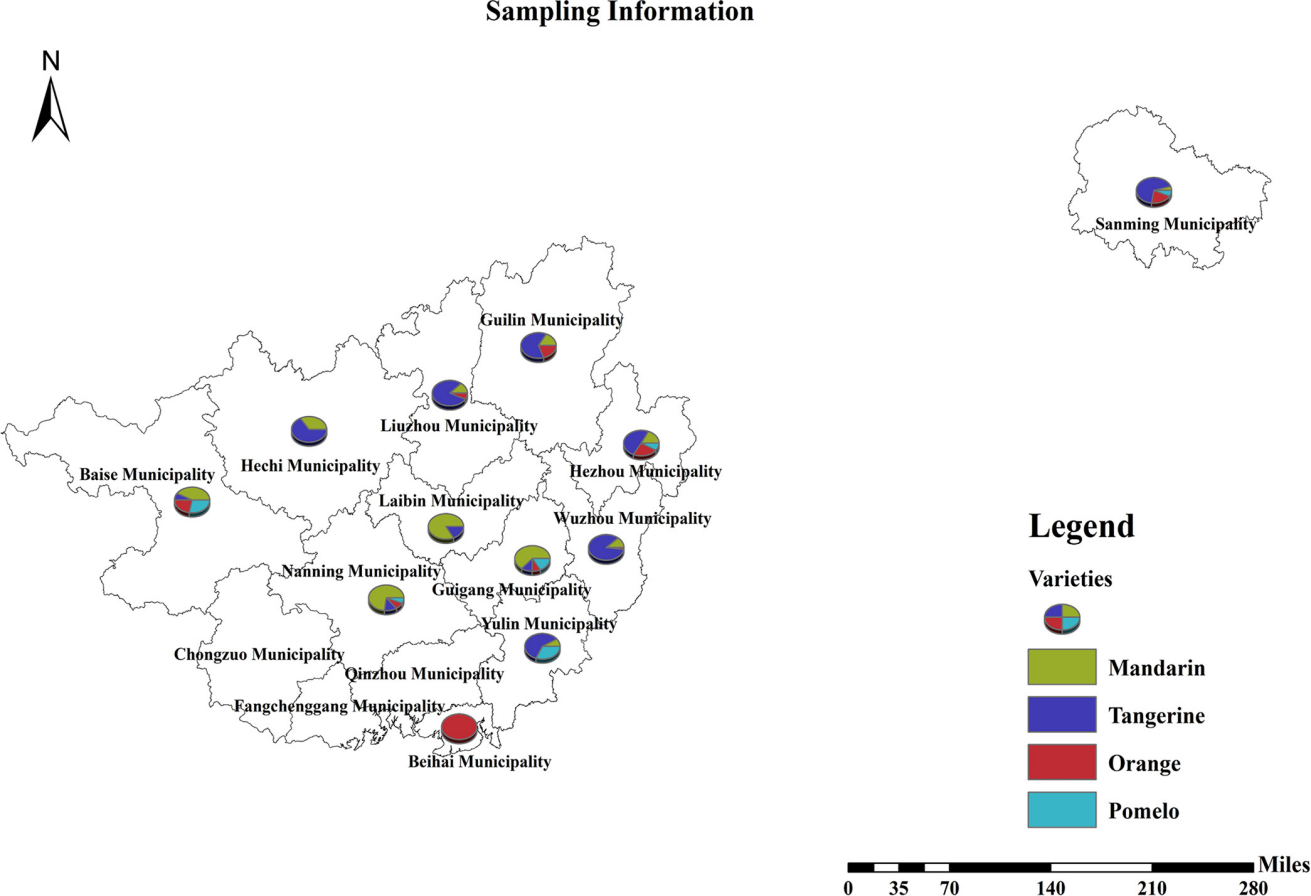
Sampling sites located in Guangxi and Fujian, China. The map was drawn with Arcgis 10.2 software.

An annual field survey was carried out in Guangxi and Fujian, China, from May to August 2020. After surveying and flagging each suspected tree by the experienced staff from the Fruit Experimental Station of the Agricultural and Rural Department of Guangxi and Fujian, 1,365 samples were randomly collected and shipped in five batches to our laboratory at Guangxi University, including 1,148 ones in four batches from eight municipalities of the Guangxi Zhuang Autonomous Region between 20 May and 20 July 2020, and 217 from two groves in Sanming municipality of Fujian Province during 15 to 16 August 2020. We also collected 423 HLB-like symptomatic leaf samples from 10 groves between September and November 2020 according to the field-sampling methods described by Irey (33), including 348 samples from seven municipalities of the Guangxi Zhuang Autonomous Region between 17 September and 16 November in five batches, and 75 ones from Sanming municipality of Fujian on 11 November 2020 (Fig. 5). The samples were picked from fully expanded leaves of the plant. Each sample consisted of 10 leaves from four sides of each tree and kept cool and out of direct sunlight, then transported to our laboratory in the cooling box containing dry ice and stored at −80°C for DNA extraction. During sampling, geographic coordinates, symptoms, and host citrus species were also recorded to maximize the coverage of *C*Las genetic diversity among citrus samples from various geographical regions and hosts.

### DNA extraction.

Genomic DNA from each sample was extracted from the leaf midrib using a modified CTAB (cetyltrimethylammonium bromide) extraction protocol as described by Allen et al. (35). The 0.1 g of leaf midribs was frozen in liquid nitrogen and quickly ground to a fine powder in the extraction buffer using FastPrep-24 Homogenizer (MP Biomedicals, LLC, OH, USA). The concentration in each sample was normalized to 25 ng/μL after quantifying with a NanoDrop spectrophotometer (Implen, CA, USA). DNA was eluted using 50 μL of TE (Tris-EDTA) buffer and stored at −20°C for further use.

### Whole-genome variation analysis for *C*Las bacteria.

The genome sequences of GXPSY (GenBank accession number: CP004005) and 34 other *C*Las isolates were downloaded from the NCBI assembly database with relatively high completeness (>1.0 Mb) (Table S1). Minimap2 v2.17 was used to align the 34 *C*Las genomes with GXPSY, and the aligned reads were sorted using Samtools v1.12 (36). BCFtools v1.12 called single nucleotide variations (including ≤50-bp indels) using the haploid model, which was also used to predict the impact of the variations on gene models (37). BEDTools v2.29 was used to analyze the presence of homologous segments and the density of SNVs and indels across the reference genome in continuous windows (38). Circos v0.69-9 was employed to plot the variation distribution across the reference (39). The nonphage and nonrepetitive genomic segments available in all 35 genomes were screened.

Biallelic variations with available genotypes were screened in all 35 genomes, and genotypes were concatenated before phylogenetic analysis. MEGA v10.2.5 was used to construct a neighbor-joining tree with 1,000 bootstrap replicates using *p*-distance (40). In Bayesian inference, we ran 1,000,000 generations of Markov chain Monte Carlo (MCMC) tree searches, sampling one tree every 1,000 generations and discarding the first 250 trees. The final phylogenetic tree was a majority-rule consensus tree in which all branches had ≥50% bootstrap support (neighbor-joining) and ≥50% posterior probability (Bayesian inference). Finally, four genomic regions with a high density of SNVs were chosen for PCR amplification and Sanger sequencing. Primer3 v0.4.0 was used to design PCR primers for the regions shown in Table 1 (41). The primers' melting temperature (*Tm*) was set at 60 ± 1°C, and the amplicon length required less than 1 kb.

### Primer design and PCR assays.

qPCR was used to detect the *C*Las bacterium in all leaf samples. Real-time PCR was used to determine the cycle threshold (*C_T_*) value by employing previously described primer sets and probes (42). Following the *C*Las-infected citrus DNA samples testing, the *C*Las-positive samples were used to amplify seven hypervariable genomic regions using the primer sets (PS) (Table 3). The qPCR procedure was performed in 25-μL mixtures containing 10 μL of 2× Rapid *Taq* Master Mix (Vazyme, China), 1 μL forward and reverse primers, 2 μL of template DNA, and 11 μL of H_2_O using a LightCycler 96 real-time PCR system (Roche, USA). The PCR cycles were programmed with an initial denaturation step of 94°C for 5 min, followed by 40 cycles of 95°C for 30 s, 56 to 60°C for 30 s, and 72°C for 30 s, based on the primer sets used. After the last cycle, a final extension of 72°C was performed for 10 min. Following confirmation, amplification products were stained with ethidium bromide under a UV illuminator on 2% agarose, purified, and sequenced using the amplification primers by Sangon Biotech (Shanghai, China).

### Genotypic and phylogenetic analysis.

Single nucleotide variations (including ≤50 bp insertion deletions) were invoked in haploid mode using Samtools v1.12 and BCFtools v1.12. Sequence identity and multiple sequence alignments were determined for all DNA sequences using the vector NTI 10. Single nucleotide polymorphism (SNP) analysis for each sample was carried out using LaunchDnaSP6 software. Specific mutation sites were estimated using Geneious R software (version 9.0.2). Unrooted neighbor-joining (NJ) was used to construct trees, and estimates of chord genetic distances were constructed using MEGA 7. The phylogenetic tree was constructed for the modes of evolutionary divergence, including the *C*Las sequences available in this study and GenBank. The *p*-distance model was used to calculate genetic diversity between populations, and the distance matrix was bootstrapped using 1,000 randomizations.

### Statistical analysis.

We calculated the pathogenic index (PI) for each location, host cultivar, or HLB-like symptom to reduce background noise in the treated trees. *C*Las bacterial titers were classified into five categories based on *C_T_* values, where category 0 = *C_T_* ≥ 36.0; category 1 = 32.0 ≤ *C_T_* < 36.0; category 2 = 28.0 ≤ *C_T_* < 32.0; category 3 = 24.0 ≤ *C_T_* < 28.0; and category 4 = *C_T_* < 24.0. For each treatment, the pathogenic index (PI) used to assess the *C*Las bacterial titer was calculated (43).

$$\text{PI}=100\;\text{*}\overset{4}{\underset{\text{n}=0}{\sum }}\frac{\text{Number of diseased plants at each level*representative value at each level}}{\text{ Number of surveyed plants }\times \text{ Highest representative value}.}$$

### Data availability.

All data are included within this article and the supplemental material. All sequences are deposited in GenBank and listed in Table S4.
