# Supplementary material for: Genetic Diversity of “Candidatus Liberibacter asiaticus” Based on Four Hypervariable Genomic Regions in China
Source: Microbiol Spectr. 2022 Nov 21;10(6):e02622-22. doi: 10.1128/spectrum.02622-22 (PMC9769890; doi:10.1128/spectrum.02622-22)
Supplement: Supplemental file 1 — Supplemental material. Download spectrum.02622-22-s0001.pdf, PDF file, 2.3 MB [file spectrum.02622-22-s0001.pdf]

Supplementary legends

Supplementary Figure S1. Gel electrophoresis of the PCR products from seven primer sets of PS1 (I), PS2 (II), PS3 (III), PS4 (IV), PS5 (V), PS6 (VI) and PS7 (VII). The amplified products from eight HLB strains were listed in lanes 3–10. Lane 1: Negative control, Lane 2: Positive control, Lane 3:BH6, Lane 4: HZ9, Lane 5:GG7, Lane 6: NN4, Lane 7:GG2, Lane 8:SM10, Lane 9: NN38, and Lane 10: NN24. Lane M: 2000 Kb ladder.

Supplementary Figure S2. Minor variation of amplified sequence from non-hypervariable genomic regions with three pairs of primer sets of PS2 (629bp), PS3 (645bp), and PS5 (670bp).

Supplementary Figure S3. Variation of the sequences of hypervariable genomic regions (HGRs) amplified by four primer sets, including PS1 (526bp), PS4 (685bp), PS6 (524bp), and PS7 (674bp).

Supplemental Table S1. Information of the 35 *C*Las genomes.

Supplementary Table S2. Single nucleotide variations and small indels in 35 *C*Las genomes.

Supplemental Table S3. Consequence of variations located in genic regions.

Supplementary Table S4. The accession numbers of the sequences amplified from seven primer sets and deposited in GenBank.

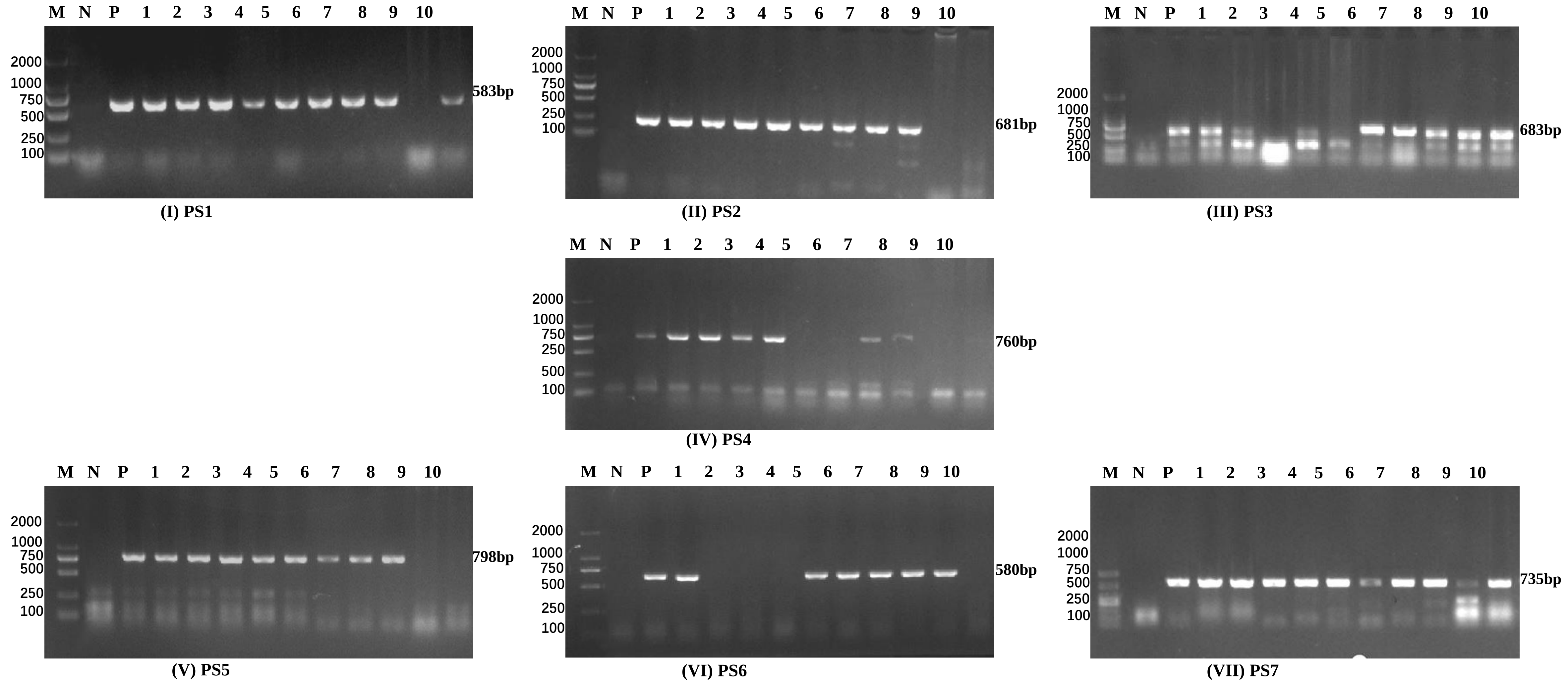

Supplementary Figure S1

PS2

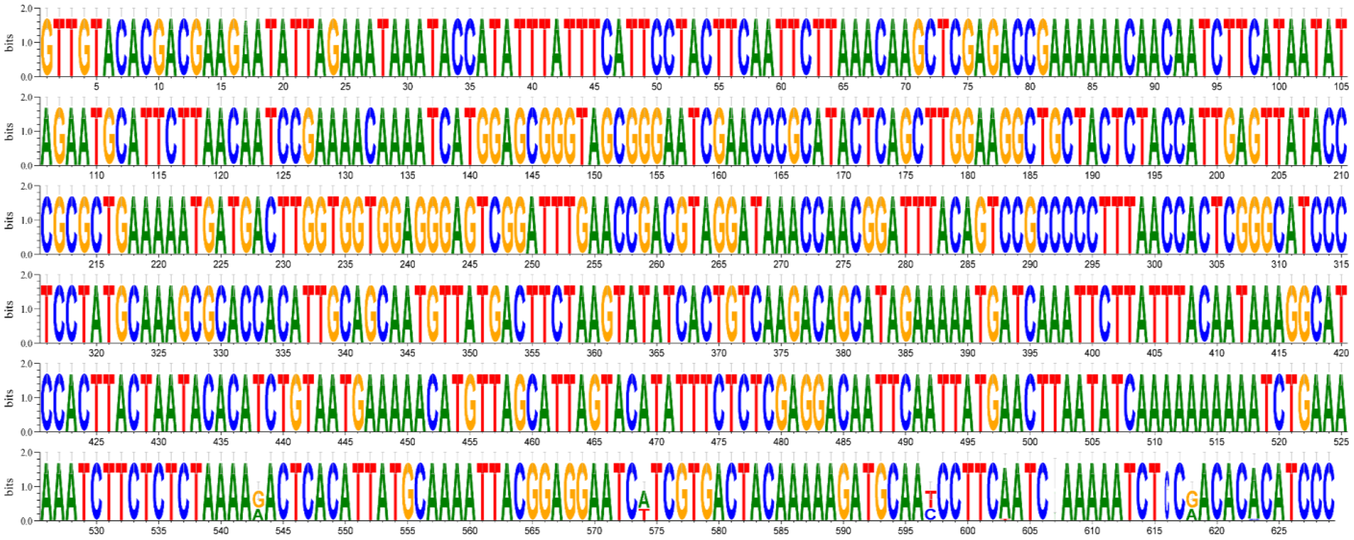

PS3

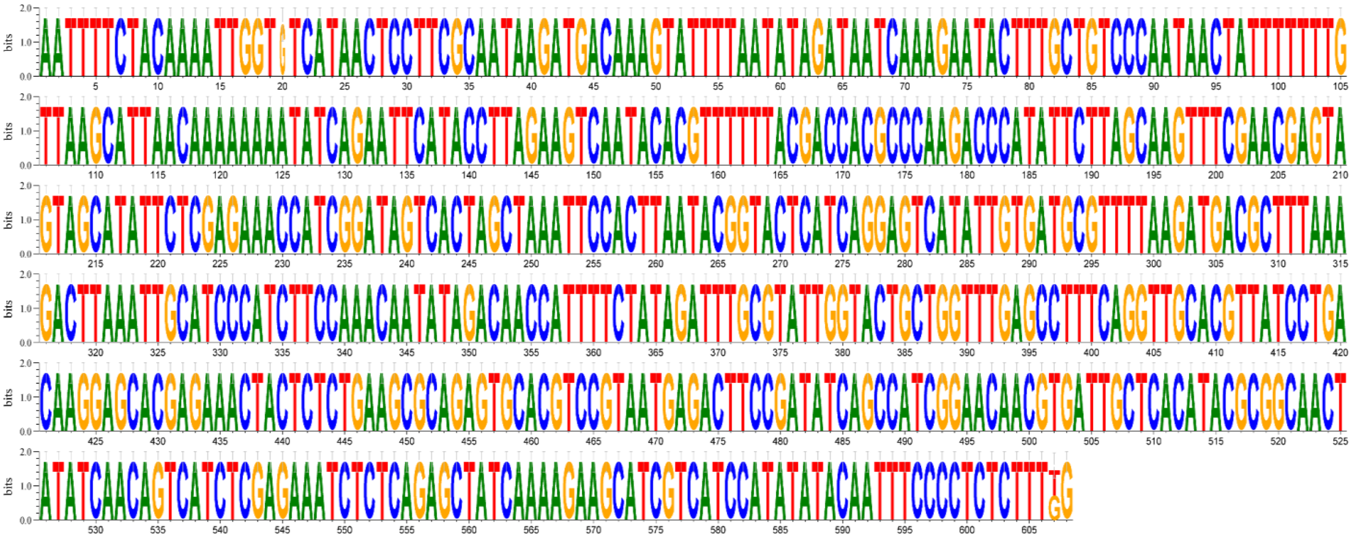

PS5

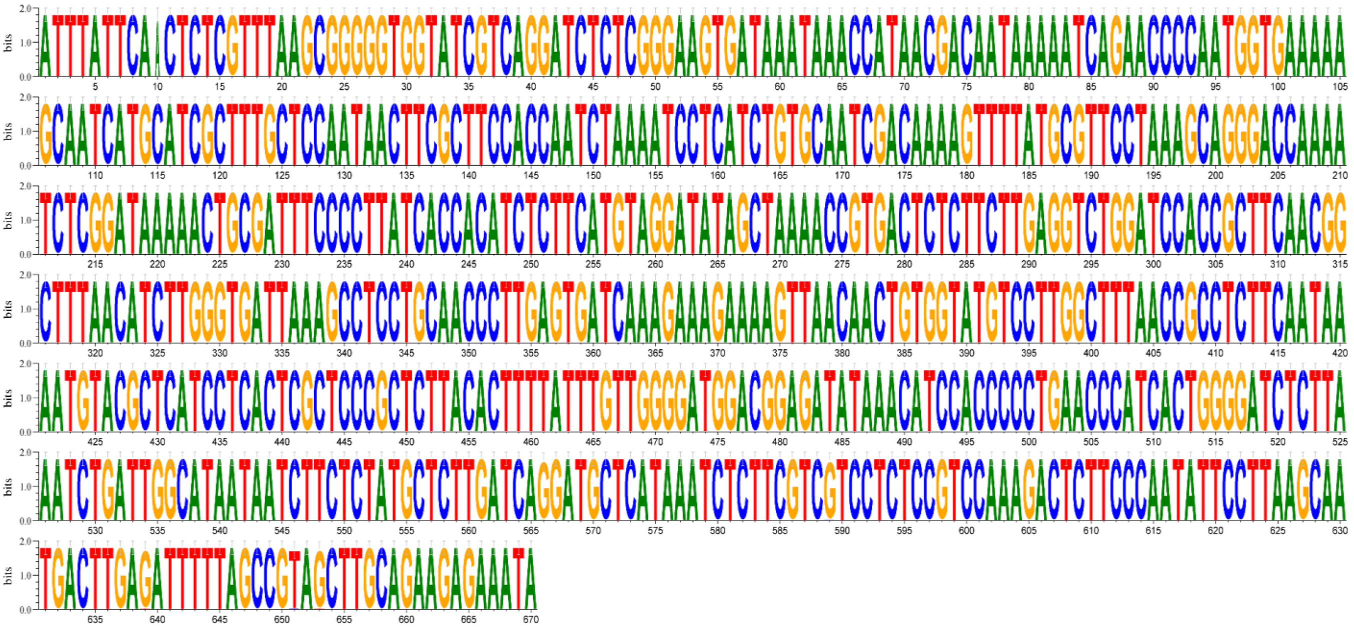

Supplementary Figure S2

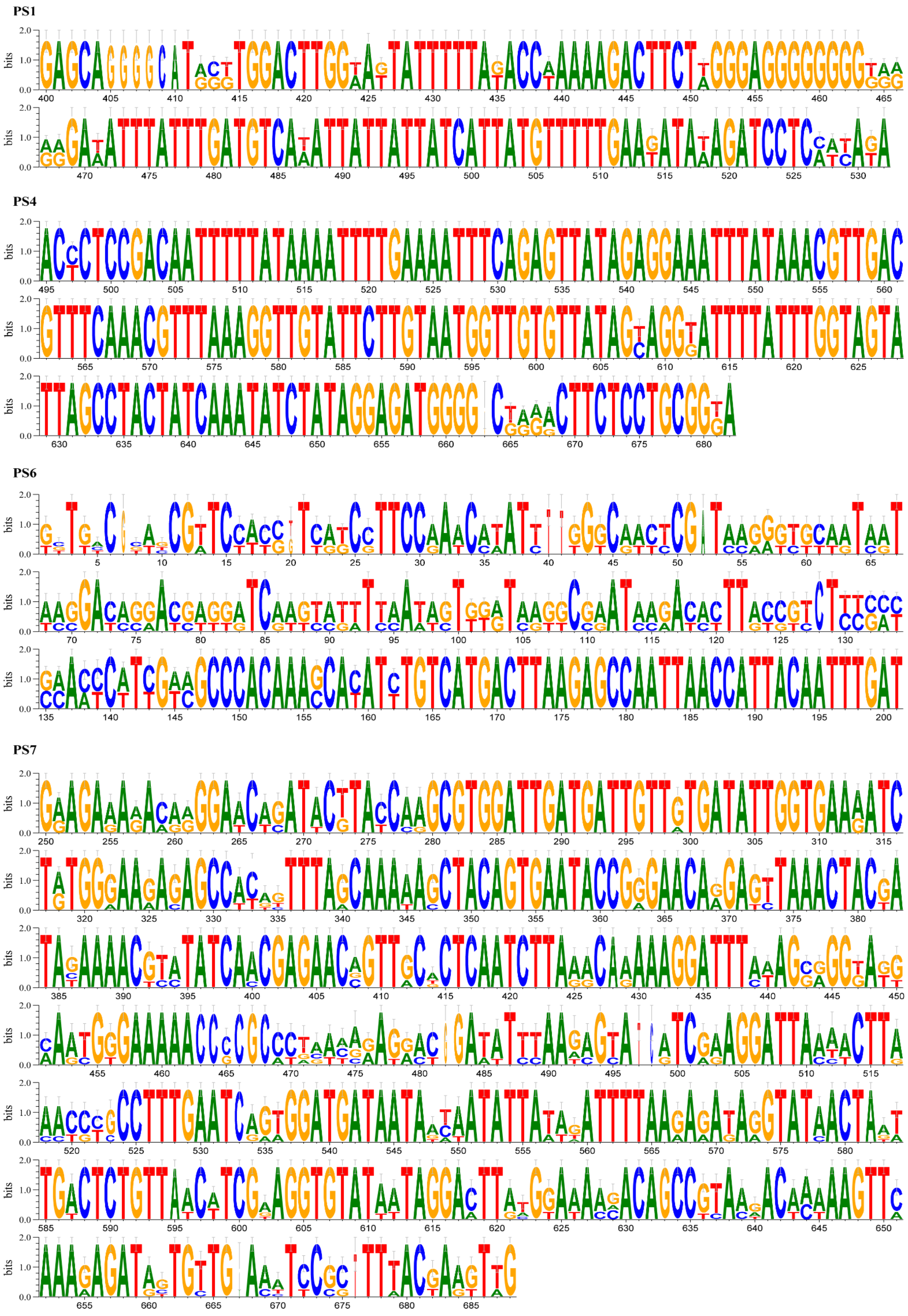

Supplementary Figure S3
